# Short-term PET-derived kinetic estimation for the diagnosis of hepatocellular carcinoma: a combination of the maximum-slope method and dual-input three-compartment model

**DOI:** 10.1186/s13244-023-01442-5

**Published:** 2023-05-24

**Authors:** Tao Wang, Boqiao Li, Hong Shi, Pengfei Li, Yinglei Deng, Siyu Wang, Qiao Luo, Dongdong Xv, Jianfeng He, Shaobo Wang

**Affiliations:** 1 Yunnan Key Laboratory of Artificial Intelligence, Faculty of Information Engineering and Automation, Kunming University of Science and Technology, Kunming, 650500 Yunnan China; 2 PET/CT Center, Affiliated Hospital of Kunming University of Science and Technology, First People’s Hospital of Yunnan, Kunming, 650031 China; 3 Yunnan Key Laboratory of Primate Biomedical Research, Institute of Primate Translational Medicine, Kunming University of Science and Technology, Kunming, China

**Keywords:** Hepatocellular carcinomas, Positron-emission tomography, Compartmental model

## Abstract

**Background:**

Kinetic estimation provides fitted parameters related to blood flow perfusion and fluorine-18-fluorodeoxyglucose (^18^F-FDG) transport and intracellular metabolism to characterize hepatocellular carcinoma (HCC) but usually requires 60 min or more for dynamic PET, which is time-consuming and impractical in a busy clinical setting and has poor patient tolerance.

**Methods:**

This study preliminarily evaluated the equivalence of liver kinetic estimation between short-term (5-min dynamic data supplemented with 1-min static data at 60 min postinjection) and fully 60-min dynamic protocols and whether short-term ^18^F-FDG PET-derived kinetic parameters using a three-compartment model can be used to discriminate HCC from the background liver tissue. Then, we proposed a combined model, a combination of the maximum-slope method and a three-compartment model, to improve kinetic estimation.

**Results:**

There is a strong correlation between the kinetic parameters *K*_1_ ~ *k*_3_, HPI and ${{\varvec{V}}}_{{\varvec{b}}}$ in the short-term and fully dynamic protocols. With the three-compartment model, HCCs were found to have higher *k*_2_, HPI and *k*_3_ values than background liver tissues, while *K*_1_, *k*_4_ and ${{\varvec{V}}}_{{\varvec{b}}}$ values were not significantly different between HCCs and background liver tissues. With the combined model, HCCs were found to have higher HPI, *K*_1_ and *k*_2_, *k*_3_ and ${{\varvec{V}}}_{{\varvec{b}}}$ values than background liver tissues; however, the *k*_*4*_ value was not significantly different between HCCs and the background liver tissues.

**Conclusions:**

Short-term PET is closely equivalent to fully dynamic PET for liver kinetic estimation. Short-term PET-derived kinetic parameters can be used to distinguish HCC from background liver tissue, and the combined model improves the kinetic estimation.

**Clinical relevance statement:**

Short-term PET could be used for hepatic kinetic parameter estimation. The combined model could improve the estimation of liver kinetic parameters.

**Graphical Abstract:**

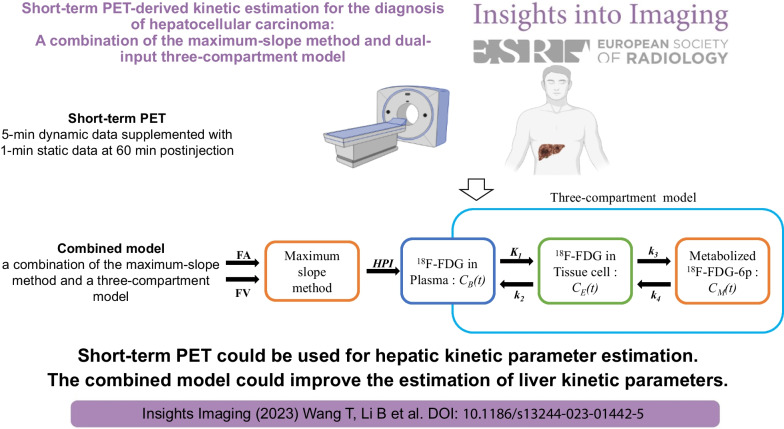

**Supplementary Information:**

The online version contains supplementary material available at 10.1186/s13244-023-01442-5.

## Key points

- Short-term PET could be used for hepatic kinetic parameter estimation.
- Short-term PET was very similar to full dynamic PET in the estimation of liver kinetic parameters.
- The combined models could improve the estimation of liver kinetic parameters.

**Supplementary Information:**

The online version contains supplementary material available at 10.1186/s13244-023-01442-5.

## Background

Hepatocellular carcinoma (HCC) is the fourth most common cause of cancer-related death worldwide. Medical imaging plays an important role in the diagnosis of HCC, increasing the chance of potentially curative treatment, overall survival, or quality of life [1].

Conventional computed tomography (CT) and magnetic resonance imaging (MR) are the primary methods used for the diagnosis of HCC, with detection rates of 60–72% and 82–95%, respectively [2, 3], but they do not provide metabolic and molecular information [4]. Since positron emission tomography (PET) or PET/computed tomography (CT), unlike other imaging modalities, visualizes tissue metabolic information, it has played a comprehensive role in detecting, distinguishing, staging, and evaluating local, residual, and recurrent HCCs [5].

However, static fluorine-18-fluorodeoxyglucose (^18^F-FDG) PET/CT has a low detection rate (between 36 and 70%) in the diagnosis of HCC [6, 7], and it only measures a single parameter, the standard uptake value (SUV), in a routine clinical setting. Kinetic estimation provides a few fitted parameters related to blood flow ^18^F-FDG transport and intracellular metabolism to characterize hepatocellular carcinoma (HCC) and is promising for accurate differentiation, therapeutic response prediction and assessment [8]. In addition, the reason for the advantage of kinetic analysis versus conventional SUV may be that kinetic data can provide an early assessment of small metabolic changes, which cannot be detected by SUV [9]. However, it usually requires 60 min or more for dynamic PET, which is time-consuming, impractical in a busy clinical setting and has poor patient tolerance.

Short-term dynamic ^18^F-FDG PET is performed synchronously with ^18^F-FDG bolus injection, scans for several minutes, and has a combination of blood flow and metabolic information [6]. Winterdahl et al. [10] demonstrated that the blood-to-cell clearance of ^18^F-FDG can be estimated by 3-min dynamic ^18^F-FDG PET/CT in healthy pigs. Samimi et al. [11] also confirmed that the use of 5-min dynamic PET data, complemented by 3-min static PET data at 60 min postinjection, allows for an accurate and robust estimation of two-compartment model parameters. Thus, this preliminary study aimed to evaluate the relevance of liver kinetic parameters between short-term (5-min dynamic data supplemented with 1-min static data at 60 min postinjection) and full 60-min dynamic protocols and whether short-term ^18^F-FDG PET-derived kinetic parameters using a three-compartment model can be used to discriminate HCC from background liver tissue.

The liver has a blood supply from both the hepatic artery and portal vein, and the weighted hepatic artery and portal vein flow forms the input function for the kinetic model. The hepatic perfusion index (HPI), as this weighted value, is crucial for kinetic estimation and significantly affects the calculation results of parameters [12, 13]. Although HPI from both the hepatic artery and portal vein can be estimated by the fitting calculation [8], the main limitation is that if one parameter is not ideally fitted, the error introduced may affect the fitting of other parameters [14]. The maximum-slope method has a relatively simple principle and is widely used in the field of hepatic blood flow estimation as well as in characterizing HCC blood flow with short-term dynamic ^18^F-FDG PET [15]. We assumed that the HPI, estimated by the maximum-slope method, calculated the input function of the dual-input three-compartment model to enhance the efficiency and accuracy of the fit calculation. Thus, we proposed a combined model, a combination of the maximum-slope method and a three-compartment model, to reduce model complexity and enhance the estimation of liver kinetic parameters.

## Methods

### Patients

From June 2022 to July 2022, a total of 27 healthy volunteers (12 males and 15 females) aged 54.4 ± 12.4 (38–80) years who received fully 60-min dynamic PET/CT scanning ^18^F-FDG PET/CT at the First People’s Hospital of Yunnan were used to validate our proposed short-term protocol, and all healthy volunteers had no history of tumor disease, chronic liver disease including hepatitis, cirrhosis or fatty liver, and normal hepatic and renal function.

From May 2020 to January 2022, a total of 21 patients (19 males and 2 females) aged 57.4 ± 17.0 (33–77) years who received 5-min dynamic ^18^F-FDG PET/CT and 60-min static ^18^F-FDG PET/CT before treatment at the First People’s Hospital of Yunnan were prospectively enrolled. Sixteen patients had cirrhosis, 19 patients had a single HCC lesion, and three patients had two HCCs; a total of 24 HCCs pathologically diagnosed by surgery (*n* = 17) or biopsy (*n* = 7) were analyzed in this study, and the long axis of these tumors was 1.9–15.0 cm (average 6.4 ± 3.7).

### Short-term and fully dynamic PET/CT

^18^F-FDG was produced in a Sumitomo HM-10HC cyclotron with a Sumitomo F300e ^18^F-FDG chemical synthesis module (Tokyo, Japan), and ^18^F-FDG had a radiochemical purity of > 95%. Scans were performed on a Philips Ingenuity TF PET/CT scanner (Cleveland, OH, USA).

Dynamic PET/CT scans are performed prior to conventional PET/CT. A bolus injection was performed with ^18^F-FDG (5.5 MBq/kg) in 2 mL of 0.9% saline and then flushed with 20 mL of 0.9% saline at a flow rate of 2 mL/s. A liver CT scan (120 kV, 100 effective mA) was performed in a single bed, and the liver was in the center of the scanner's field of view. A list mode of dynamic PET of the liver scan was performed concurrently with the administration of the ^18^F-FDG bolus. All healthy volunteers were scanned for 60 min in full dynamic PET, reconstructed as 37 consecutive time frames (12 × 5 s, 9 × 60 s, 10 × 120 s, 6 × 300 s). All HCC patients were scanned for 5 min of dynamic PET, reconstructed as 16 consecutive time frames (12 × 5 s, 4 × 60 s).

Routine static scans were performed approximately 60 min after the ^18^F-FDG bolus, including from the vertex of the skull to the proximal thigh, with 1 min of scanning in each bed.

**Fig. 1:**
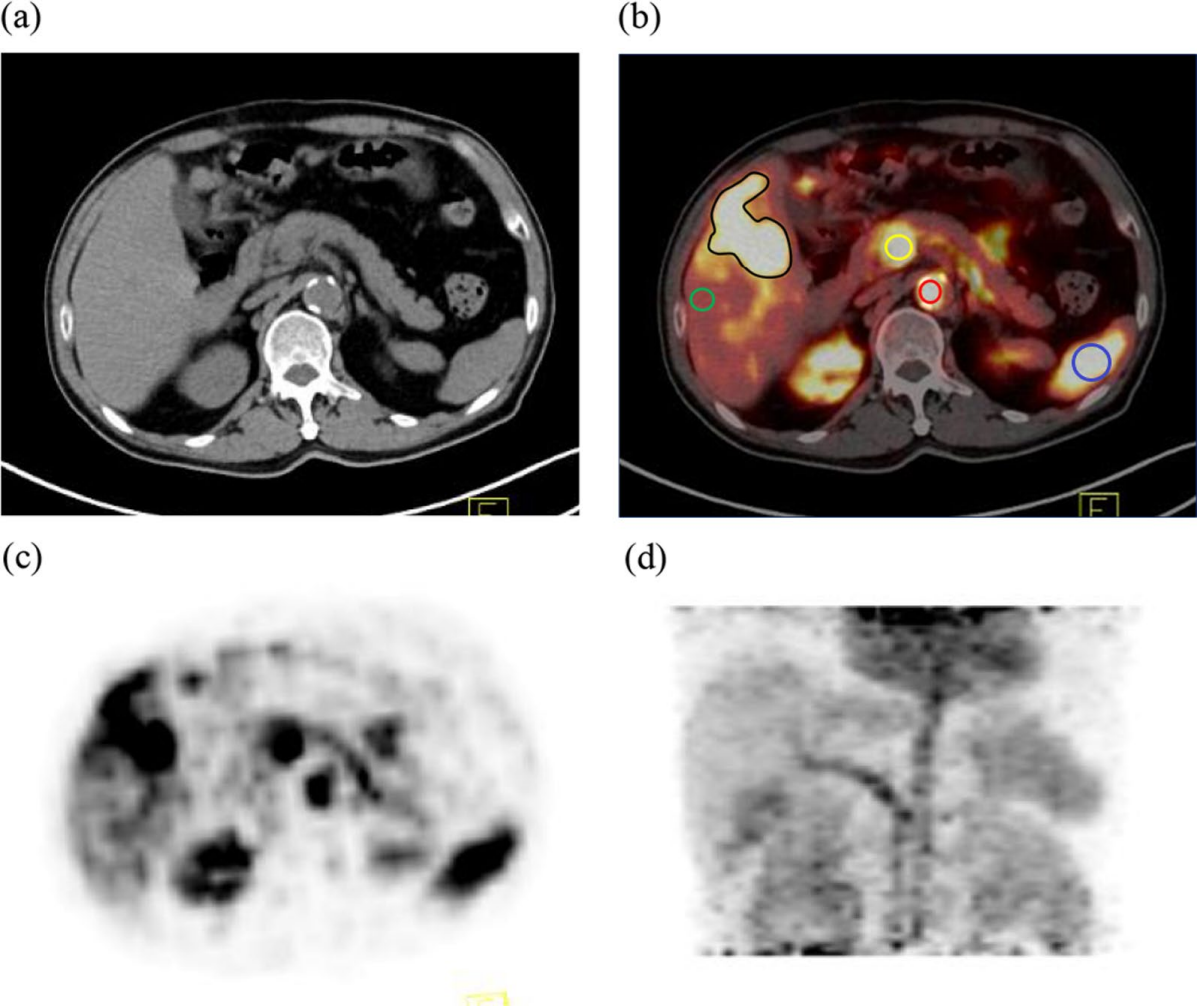
Delineating regions of interest (ROIs) in dynamic PET/CT. **a** CT image, (**b**) PET/CT image, (**c**) PET image, and (**d**) maximum density projection image. ROIs are manually drawn, and HCC is shown in black, background liver tissues in green, aorta in red, portal vein in yellow and spleen in blue

The SUVmax was measured from PET images by delineating 2D circular regions of interest (ROIs) (Fig. 1), and the ROIs were drawn by Dr. Shaobo Wang and Dr. Shiyu Wang, two nuclear radiologists. In those lesions with imperceptible FDG uptake, ROIs were drawn relative to the conventional imaging findings. ROIs of the aorta and portal vein were placed at approximately two-thirds of the vascular cross section. To compare HCC tumors to the background tumor-free liver tissue, the respective ROIs were drawn in tumor-free liver tissue, and all ROIs avoided blood vessels. Eighty-one ROIs of liver tissue from 27 healthy volunteers (three ROIs in each healthy volunteer) and 24 pathologically diagnosed HCCs and 21 healthy liver regions were delineated from 21 patients.

### Three-compartment model

**Fig. 2:**
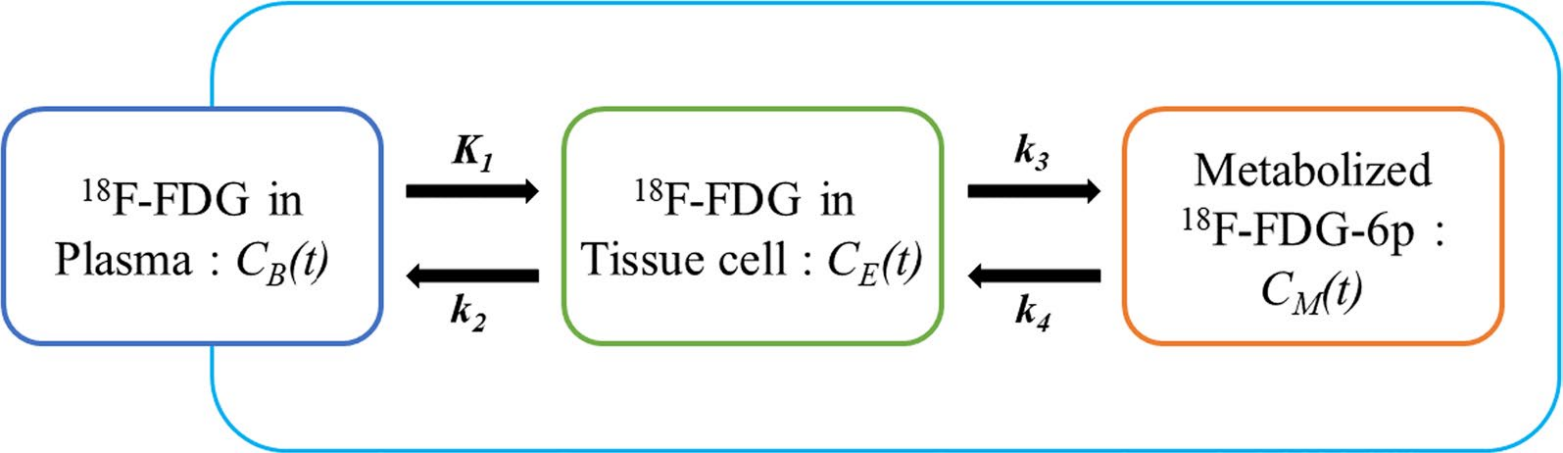
Reversible three-compartment model for ^18^F-FDG kinetics

In the three-compartment hemodynamic model, the glucose passing rate constant *K*_1_ (mL/min/mL) and the opposite direction passing rate constant *k*_2_ (1/min) represent glucose transport from the blood to the liver tissues. The process by which hexokinase further phosphorylates ^18^F-FDG to ^18^F-FDG-6-phosphate is represented by another rate constant, *k*_*3*_ (1/min), and the dephosphorylation rate constant *k*_*4*_ (1/min). Since liver tissues and tumors have different intake and metabolic rates for ^18^F-FDG, the corresponding kinetic parameters can be obtained only when the model is calculated [16]. These processes are the basis of the hypothetical model, and the curve is equivalent to the background liver tissue or HCC time-activity curve (TAC) measured in the PET image [17]. In Fig. 2, the left compartment represents the blood space, the middle compartment represents ^18^F-FDG in the tissue, the right compartment represents the product ^18^F-FDG-6-p after ^18^F-FDG is phosphorylated in the tissue, *C*_B_(*t*), *C*_E_(*t*), and *C*_M_(*t*) represent the concentrations of ^18^F-FDG or ^18^F-FDG 6-p in these three compartments, respectively, and the kinetic parameters *K*_1_–*k*_4_ represent the rate coefficients of the material exchange between the compartments.

This article uses a dual blood supply model, and the total input blood volume is expressed by *C*_B_*(t)*, which is the input function of the model, as shown in Formula 1:

1 $$\begin{array}{*{20}c} {C_{{\text{B}}} \left( t \right) = {\text{HPI}} \times A\left( t \right) + \left( {1 - {\text{HPI}}} \right) \times V\left( {\text{t}} \right)} \\ \end{array}$$

where *A(t)* represents the ^18^F-FDG concentration in the hepatic artery, *V(t)* represents the ^18^F-FDG concentration in the portal vein, and HPI represents the hepatic artery perfusion index (the ratio of arterial blood volume to total blood volume) [18].

2 $$\begin{array}{*{20}c} {C_{{\text{T}}} \left( {\text{t}} \right) = \left( {1 - V_{{\text{b}}} } \right) \times C_{{\text{I}}} \left( t \right) + V_{{\text{b}}} \times C_{{\text{B}}} \left( t \right)} \\ \end{array}$$

3 $$\begin{array}{*{20}c} {C_{I} = C_{E} \left( t \right) + C_{M} \left( t \right)} \\ \end{array}$$

*C*_T_*(t)* is equivalent to the curve of the tracer concentration in the tissue observed from the PET image over time, and ${V}_{\mathrm{b}}$ (unitless) represents the fraction of the measured volume occupied by blood [18]. Through the calculation of the three-compartment model, Formula 4 can be obtained [18, 19]:

4 $$\begin{array}{*{20}c} {C_{{\text{T}}} \left( {\text{t}} \right) = \left( {1 - V_{{\text{b}}} } \right) \times \frac{{K_{1} }}{{T_{2} - T_{1} }} \times \left[ {\left( {k_{3} + k_{4} - T_{1} } \right)e^{{ - T_{1} t}} + \left( {T_{2} - k_{3} - k_{4} } \right)e^{{ - T_{2} t}} } \right] \otimes C_{{\text{B}}} \left( t \right) + V_{{\text{b}}} \times C_{B} \left( t \right)} \\ \end{array}$$

where ⨂ represents the convolution operation. Formulas 5–6 are shown below:

5 $$\begin{array}{*{20}c} {T_{1} = \frac{{k_{2} + k_{3} + k_{4} - \sqrt {\left( {k_{2} + k_{3} + k_{4} } \right)^{2} - 4k_{2} \times k_{4} } }}{2}} \\ \end{array}$$

6 $$\begin{array}{*{20}c} {T_{2} = \frac{{k_{2} + k_{3} + k_{4} + \sqrt {\left( {k_{2} + k_{3} + k_{4} } \right)^{2} - 4k_{2} \times k_{4} } }}{2}} \\ \end{array}$$

### Maximum-slope method model

The maximum-slope method presented by Mullani et al. [20, 21] was used to calculate the required blood flow parameters [22]. The model assumes that when the tracer first passes through the tissue, the venous exit of the tracer is delayed for a period of time, which is a function of the volume of distribution of the tracer in the target tissue and the blood vessel density [23]. During this time delay, for highly extracted tracers, most of the tracers remain in the tissue because the vein outlet is very small [24]. The liver has a dual blood supply of arteries and portal veins. The unit for calculated hepatic blood flow is mL/mL/s, which should be converted to mL/100 mL/min.

The blood flow parameters included hepatic artery perfusion (HAP), hepatic vein perfusion (HVP), total liver perfusion (TLP) and HPI.

The basic formulas (7–10) of the above parameters are as follows:

7 $$\begin{array}{*{20}c} {{\text{HAP}} = \frac{{S_{{{\text{ART}}}} }}{{A_{{{\text{ART}}}} }} \times 6000} \\ \end{array}$$

8 $$\begin{array}{*{20}c} {{\text{HVP}} = \frac{{S_{{{\text{PV}}}} }}{{A_{{{\text{PV}}}} }} \times 6000} \\ \end{array}$$

9 $${\text{TLP}} = {\text{HAP}} + {\text{HVP}}$$

10 $$\begin{array}{*{20}c} {{\text{HPI}} = \frac{{{\text{HAP}}}}{{{\text{HAP}} + {\text{HVP}}}}} \\ \end{array}$$

In these formulas, *S*_ART_ represents the maximum slope of the TAC of background liver tissues (or tumor tissues) before the peak of splenic parenchymal enhancement, *S*_PV_ is the maximum slope of the TAC of background liver tissues (or tumor tissues) after the peak of splenic parenchymal intensity, ${A}_{\mathrm{ART}}$ represents the peak of abdominal aortic intensity, and ${A}_{\mathrm{PV}}$ represents the peak of portal vein intensity [25].

**Fig. 3:**
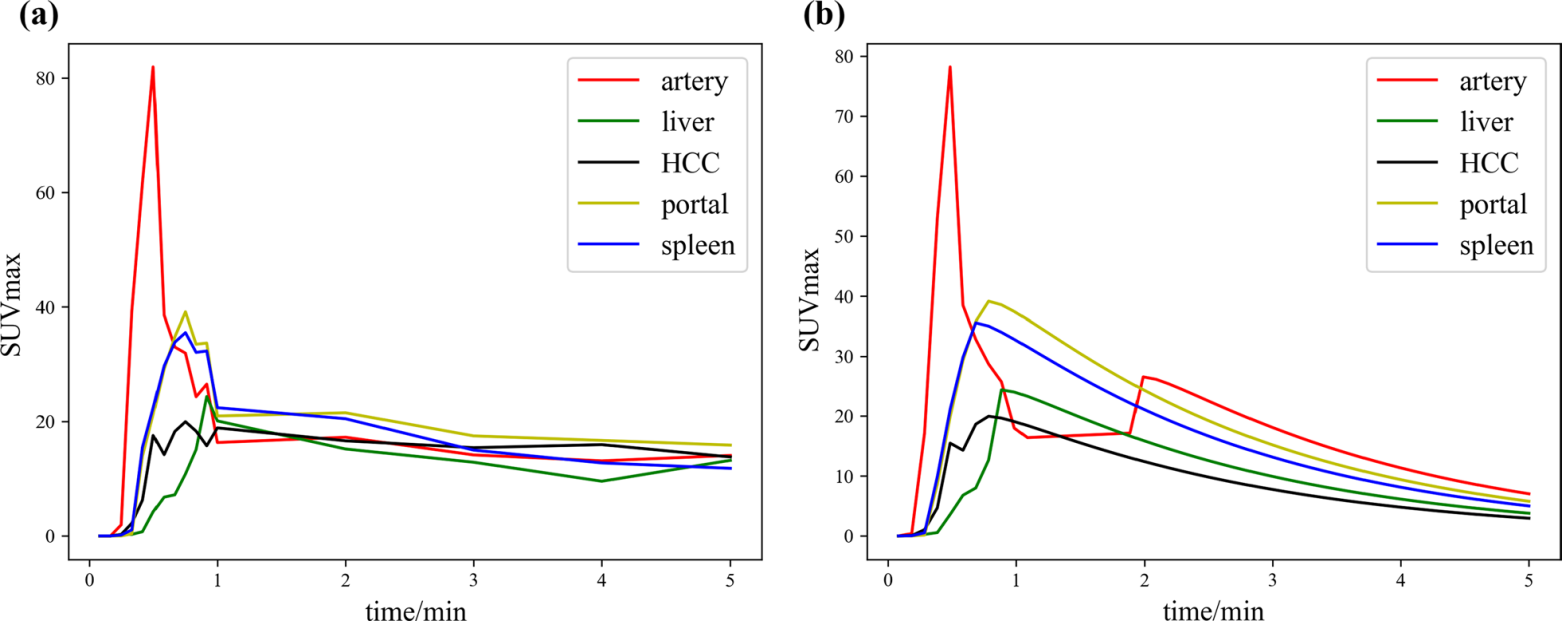
HCC time-activity curves (TACs) at 5 min. **a** Original TACs, **b** TACs after gamma correction

Due to arterial recirculation, secondary blood supply will produce secondary peaks, which will interfere with the calculations [15]. Therefore, the gamma variable is added, and gamma variable fitting is used to correct arterial recirculation and determine the peak tissue activity of noisy samples [26]. The basic gamma variable function is defined as Formula 11.

11 $$\begin{array}{*{20}c} {Y\left( t \right) = At^{\alpha } \exp \left( { - \frac{t}{\beta }} \right),\quad t > 0} \\ \end{array}$$

where $A = y_{\max } t_{\max }^{ - \alpha } \exp \left( \alpha \right)$, and $\beta = \frac{{t_{\max } }}{\alpha }$ [15]. This expression can also be expressed as Formula 12:

12 $$\begin{array}{*{20}c} {Y\left( t \right) = y_{\max } \left( {\frac{t}{{t_{\max } }}} \right)^{\alpha } \exp \left[ {\alpha \left( {1 - \frac{t}{{t_{\max } }}} \right)} \right],\quad t > 0} \\ \end{array}$$

where $y_{\max }$ is the maximum value and $t_{\max }$ is the time to the maximum value. The TACs before and after the correction are shown in Fig. 3. The time to peak (TTP) can identify background liver tissue from HCC because HCC tumors are primarily nourished by arterial blood flow, and the arterial blood flow peaks earlier than the portal blood flow at the first pass [15]:

13 $$\begin{array}{*{20}c} {{\text{TTP}} = t_{\max } - t_{{\text{a}}} } \\ \end{array}$$

where $t_{\max }$ is the time for the ROI to peak and $t_{{\text{a}}}$ is the time for the aorta to peak.

### Combined model

**Fig. 4:**
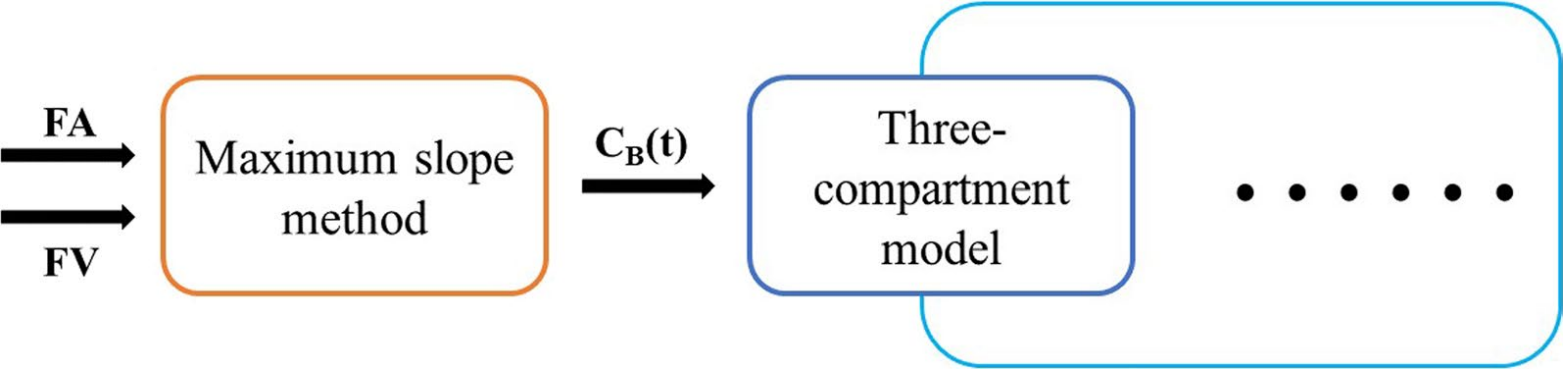
The combined model of ^18^F-FDG kinetics. The arterial blood flow and venous blood flow were first calculated by the maximum-slope method and then entered into the three-compartment model for fitting calculation

A combined model based on the three-compartment model, replacing the original HPI (a parameter to be fitted) with the HPI calculated using the maximum-slope model, is proposed in this study (Fig. 4). We expect that replacing the important parameter HPI with more accurate calculated values and then calculating the three-compartment model can improve the accuracy of other parameters. The dual blood supply in the three-compartment model will be calculated with Formula 14:

14 $$\begin{array}{*{20}c} {C_{{\text{B}}} \left( t \right) = \frac{{\frac{{S_{{{\text{ART}}}} }}{{A_{{{\text{ART}}}} }}}}{{\frac{{S_{{{\text{ART}}}} }}{{A_{{{\text{ART}}}} }} + \frac{{S_{{{\text{PV}}}} }}{{A_{{{\text{PV}}}} }}}} \times 100\% \times A\left( t \right) + \left( {1 - \frac{{\frac{{S_{{{\text{ART}}}} }}{{A_{{{\text{ART}}}} }}}}{{\frac{{S_{{{\text{ART}}}} }}{{A_{{{\text{ART}}}} }} + \frac{{S_{{{\text{PV}}}} }}{{A_{{{\text{PV}}}} }}}} \times 100\% } \right) \times V\left( t \right)} \\ \end{array}$$

### Parameter estimation

Combined model and three-compartment model fitting were performed according to the nonlinear least square method, and the unknown model parameters were estimated by iteratively fitting the model output function *C*_T_(*t*) and *c* of the PET measurements and implemented using MATLAB, R2019a (MathWorks, Natick, MA, USA). The unknown model parameter set of the combined model is *θ* = $\left[{K}_{1},{k}_{2}, {k}_{3}, {k}_{4}, {V}_{b}\right]$, and the three-compartment model is *θ* = $\left[{K}_{1},{k}_{2}, {k}_{3}, {k}_{4}, \text{HPI}, {V}_{b}\right]$:

15 $$\begin{array}{*{20}c} {\hat{\theta } = \mathop {\mathrm{argmin}}\limits_{\theta } WRSS\left( \theta \right)} \\ \end{array}$$

16 $$\begin{array}{*{20}c} {{\text{WRSS}}\left( \theta \right) = \mathop \sum \limits_{i = 1}^{N} w_{i} \left[ {c_{i} - C_{T} \left( {t_{i} ;\theta } \right)} \right]} \\ \end{array}$$

where WRSS(θ) represents the weighted residual sum of squares of the curve fit, and ${w}_{i}$ represents the weighting factor of time frame *N*.

### Statistical analysis

Statistical analysis was performed using MedCalc version 13.0.0.0 (MedCalc Software, Ostend, Belgium). Pearson (*r*) was used to assess the correlation between the kinetic parameters calculated by the short-term and fully dynamic protocols in healthy volunteers, and Passing‒Bablok regressions were used to test for differences between the kinetic parameters calculated by the two acquisition protocols [27]. Using receiver operating characteristic curve analysis, the kinetic parameters between HCCs and background liver tissues were compared in HCC patients, and kinetic parameters in the liver tissue were compared between HCC patients and healthy volunteers. *p* < 0.05 indicated significant differences. The median, mean and standard deviation of kinetic parameters in the three-compartment model and combined model are shown by box plots.

The TAC fit quality between the three-compartment model and the combined model was compared using the goodness of fit (*R*) for nonlinear regression [28, 29]:

17 $$\begin{array}{*{20}c} {R = 1 - \sqrt {\frac{{\sum \left( {y - \hat{y}} \right)^{2} }}{{\sum y^{2} }}} } \\ \end{array}$$

where $y$ represents the original data and $\widehat{y}$ represents the fitted data.

## Results

### Kinetic estimation between short-term and fully dynamic protocols

The results of the correlation and Passing–Bablok analyses between kinetic parameters calculated using fully dynamic and short-term PET/CT data from 27 healthy volunteers are shown in Table 1.

**Table 1:** Correlation and regression analysis between kinetic parameters for short-term and fully dynamic protocols

Parameters	Pearson		Passing–Bablok	
	r (95% CI)	*p*	Intercept (95% CI)	Residual SD (95% CI)
*K*_1_	0.984 (0.975 to 0.990)	< 0.001	0.182 (0.059 to 0.314)	0.166 (− 0.326 to 0.326)
*k*_2_	0.919 (0.877 to 0.947)	< 0.001	0.074 (− 0.021 to 0.152)	0.133 (− 0.260 to 0.260)
*k*_3_	0.748 (0.631 to 0.830)	< 0.001	0.012 (0.006 to 0.015)	0.014 (− 0.026 to 0.026)
*k*_4_	0.258 (0.182 to 0.445)	0.020	0.039 (0.027 to 0.056)	0.028 (− 0.054 to 0.054)
HPI	0.965 (0.947 to 0.978)	< 0.001	− 0.001 (− 0.003 to 0.001)	0.028 (− 0.055 to 0.055)
${V}_{\mathrm{b}}$	0.977 (0.965 to 0.985)	< 0.001	− 0.001 (− 0.001 to 0.001)	0.007 (− 0.013 to 0.013)

The kinetic parameters *K*_1_–*k*_3_, HPI and ${{\varvec{V}}}_{{\varvec{b}}}$ were strongly correlated (*r* ≥ 0.748, *p* < 0.05), and Passing–Bablok regression analysis showed no significant bias. However, *k*_*4*_ was weakly correlated (*r* = 0.258, *p* = 0.020).

### Three-compartment model parameters

The ^18^F-FDG PET-derived kinetic parameters obtained using the three-compartment model for 21 HCC patients are shown in Table 2. HCCs showed higher *k*_2_ (*p* = 0.048), *k*_3_ (*p* = 0.030) and HPI (*p* < 0.001) than the background liver tissues.

**Table 2:** ^18^F-FDG PET-derived kinetic parameters for HCCs and the background liver tissues by using the three-compartment model

Parameters	HCCs *n* = 24	Liver tissue *n* = 21	AUC	*p*
HPI (%)	71.14 ± 24.56	16.78 ± 20.29	0.944	< 0.001
*K*_1_ (mL/min/mL)	1.67 ± 0.52	1.65 ± 0.73	0.511	0.927
*k*_2_ (1/mL)	2.01 ± 0.43	1.66 ± 0.73	0.633	0.048
*k*_3_ (1/mL)	0.051 ± 0.050	0.013 ± 0.021	0.784	0.003
*k*_4_ (1/mL)	0.019 ± 0.027	0.023 ± 0.036	0.524	0.706
${V}_{\mathrm{b}}$ (unitless)	0.081 ± 0.138	0.027 ± 0.056	0.649	0.098
SUVmax	5.320 ± 2.971	2.505 ± 0.629	0.859	< 0.001

Compared with the background liver tissues, the *K*_1_ and ${V}_{\mathrm{b}}$ values of HCCs were increased, but these differences were not statistically significant (*p* = 0.927 and *p* = 0.098). Moreover, the value of *k*_4_ was not significantly different between HCCs and the background liver tissues (*p* = 0.098).

The SUVmax was higher in HCCs than in the background liver tissues (*p* < 0.001).

### Maximum-slope model parameters

The ^18^F-FDG PET-derived parameters obtained using the maximum-slope model for 21 HCC patients are shown in Additional file 1: Table S1. HCCs showed higher HAP (*p* < 0.001) and TLP (*p* < 0.001) than the background liver tissues. The HVP was not significantly different between HCCs and the background liver tissues (*p* = 0.462), and the TTP of HCCs was lower than that of the background liver tissues (*p* = 0.002).

### Combined model parameters

The HPI was calculated from the maximum-slope method, and the ^18^F-FDG PET-derived parameters obtained using the combined model for 21 HCC patients are shown in Table 3. HCCs showed higher HPI (*p* < 0.001), *K*_1_ (*p* = 0.007), *k*_2_ (*p* < 0.001), *k*_3_ (*p* < 0.001), and ${V}_{\mathrm{b}}$ (*p* < 0.001) values than the background liver tissues.

**Table 3:** ^18^F-FDG PET-derived kinetic parameters of HCCs and background liver tissues by using the combined model

Parameters	HCC *n* = 24	Liver tissues *n* = 21	AUC	*p*
HPI (%)	76.43 ± 12.09	42.76 ± 17.08	0.937	< 0.001
*K*_1_ (mL/min/mL)	1.52 ± 0.61	1.00 ± 0.63	0.750	0.007
*k*_2_ (1/mL)	1.84 ± 0.56	1.01 ± 0.59	0.843	< 0.001
*k*_3_ (1/mL)	0.046 ± 0.047	0.005 ± 0.007	0.866	< 0.001
*k*_4_ (1/mL)	0.021 ± 0.027	0.023 ± 0.034	0.530	0.896
${V}_{\mathrm{b}}$ (unitless)	0.052 ± 0.053	0.003 ± 0.007	0.810	< 0.001

However, *k*_4_ was not significantly different between HCCs and the background liver tissues (*p* = 0.896).

### Kinetic parameters between HCC patients and healthy volunteers

The ^18^F-FDG PET-derived kinetic parameters obtained using the combined model of liver tissues from 21 HCC patients and 27 healthy volunteers are shown in Table 4. The liver tissues of HCC patients showed a higher HPI (*p* < 0.001) than those of healthy volunteers. Healthy volunteers had higher *K*_1_ (*p* = 0.020), *k*_2_ (*p* = 0.026), *k*_3_ (*p* = 0.002) and ${V}_{\mathrm{b}}$ (*p* < 0.001) in liver tissues than HCC patients.

**Table 4:** ^18^F-FDG PET-derived kinetic parameters obtained using the combined model of liver tissues between HCC patients and healthy volunteers

Parameters	Cirrhosis *n* = 21	Normal liver tissue *n* = 81	AUC	*p*
HPI (%)	42.76 ± 17.08	18.02 ± 11.52	0.958	< 0.001
*K*_1_ (mL/min/mL)	1.00 ± 0.63	1.36 ± 0.40	0.716	0.020
*k*_2_ (1/mL)	1.01 ± 0.59	1.37 ± 0.44	0.685	0.026
*k*_3_ (1/mL)	0.005 ± 0.007	0.030 ± 0.036	0.815	0.002
*k*_4_ (1/mL)	0.023 ± 0.034	0.036 ± 0.037	0.591	0.154
${V}_{\mathrm{b}}$ (unitless)	0.003 ± 0.007	0.025 ± 0.065	0.961	< 0.001

However, *k*_4_ was not significantly different between the liver tissues of HCC patients and healthy volunteers (*p* = 0.154).

### Changes in kinetic parameters

**Fig. 5:**
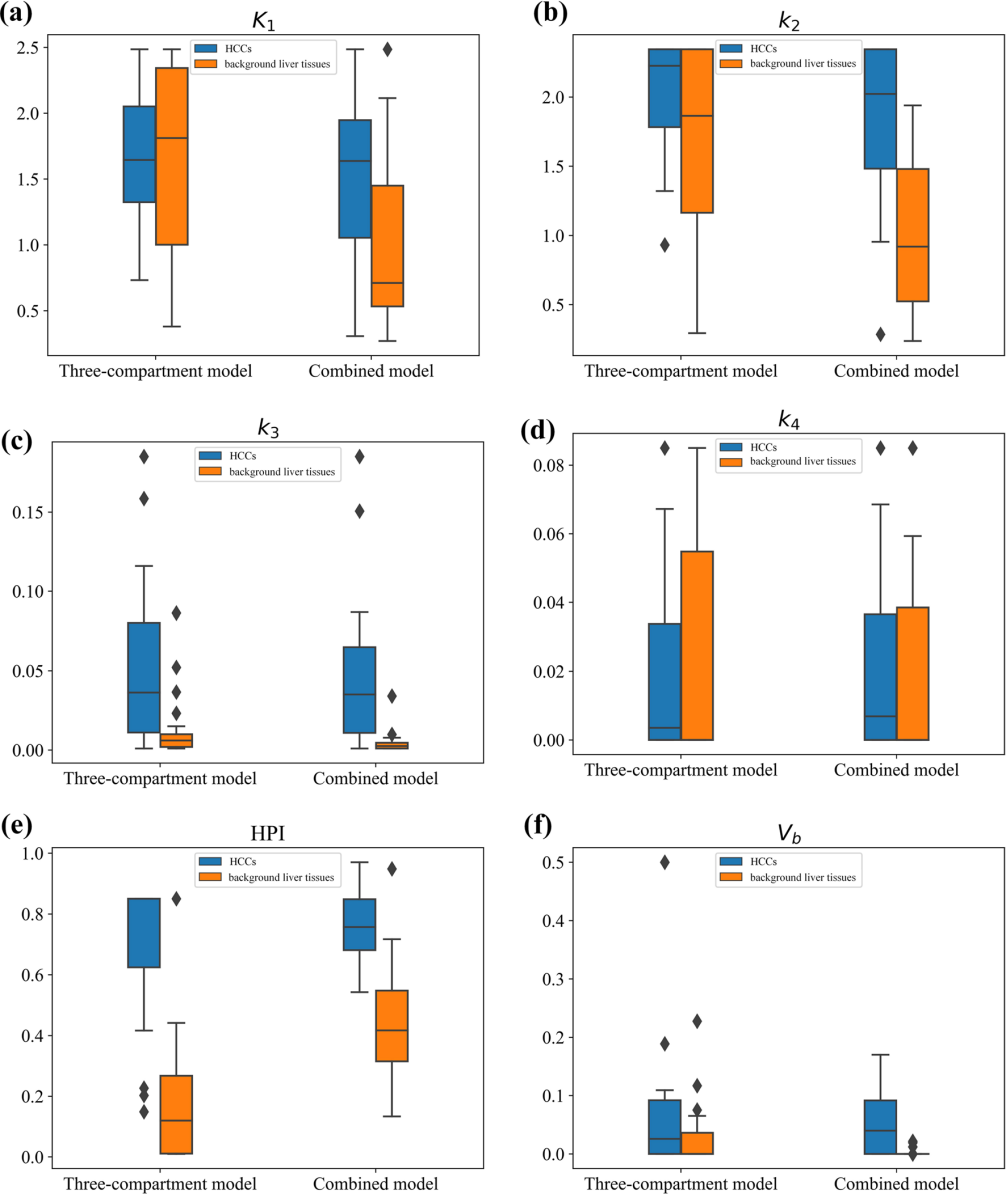
Box plots of kinetic parameters in the three-compartment model and the combined model. The diamond indicates the outliers. **a** Box plot of *K*_1_, **b** Box plot of *k*_2_, **c** Box plot of *k*_3_, **d** Box plot of *k*_4_, **e** Box plot of HPI, **f** Box plot of *V*_b_,

Figure 5 shows the box plot of kinetic parameters for the three-compartment model and combined model. In both HCCs and background liver tissues, *K*_1_, *k*_3_, HPI and ${V}_{\mathrm{b}}$ in the combined model showed a more compact data distribution, smaller standard deviation, and stronger consistency between the mean and median.

### TAC fit quality

The goodness of fit of the two models (three-compartment model and combined model) is shown in Table 5. The quality of fit of the combined model was better than that of the three-compartment model in the background liver tissues (*p* = 0.005). For HCCs, the quality of fit of the two models was comparable (*p* = 0.231).

**Table 5:** The goodness of fit of the two models

Model	HCCs	Liver tissues
	*n* = 24	*n* = 21
Three-compartment model	81.58 ± 22.58	87.68 ± 11.07
Combined model	84.49 ± 23.18	91.43 ± 7.15
*p*	0.231	0.005

## Discussion

To address the problem that 60-min or more dynamic PET is not suitable for busy clinical settings and has poor patient tolerance, some researchers have begun to explore short-term dynamic PET to assess hemodynamics. Winterdahl et al. [10] demonstrated that 3-min dynamic PET data can be used to estimate liver blood flow in pigs. By using the maximum-slope method, Bernstine et al. [15] demonstrated that 1.5-min dynamic PET-derived blood flow parameters can be used to help distinguish and characterize HCCs. They found that HPI showed better performance in distinguishing HCCs from background liver tissues than SUVmax using conventional PET. Samimi et al. [11] also found that 5-min dynamic with static PET/CT data after 60 min can be analyzed using the dual-compartment model and showed a strong correlation between all kinetic parameters from 60-min full scanning in myocardium, normal lung and lung tumor. However, the liver kinetics were not evaluated with a short-term dynamic PET protocol, and/or the kinetic model was relatively simple. This study preliminarily evaluated the feasibility of a 5-min dynamic combined with 1-min static at 60 min postinjection PET in liver kinetics with a dual-input three-compartment model. Compared with static PET/CT SUVmax, kinetic parameters derived by three-compartment models enable better distinction between HCC and background liver tissue, and this paper proposes a combination of the maximum-slope method and the three-compartment model that was preliminarily introduced to enhance the fitting calculation.

Based on the findings of previous studies and our clinical practice [6], short-term PET data (5-min dynamic data complemented by 1-min static data at 60 min postinjection) were used in this study. Although the ideal sampling for the fitting calculation is to divide the scanning time to be as short as possible, the signal-to-noise ratio (SNR) decreases significantly. This paper selects SUVmax to form the TAC for dynamics modeling. According to our clinical practice and prior literature, SUVmax has better repeatability than SUVmean because it does not depend heavily on the delineation of the ROI [30–32]. Additionally, SUVmax changed greatly in the first 1 min and then fluctuated slightly and became flat (Fig. 3a); thus, 5-s data for each frame in the first 1 min and 60-s data for each frame in the following 4 min were reconstructed in this study, and static images were taken 60 min after injection. To validate our proposed short-term acquisition protocol, 30 healthy volunteers were recruited for a 60-min fully dynamic scan. The results showed that there were strong correlations between the kinetic parameters *K*_1_–*k*_3_, HPI and ${{\varvec{V}}}_{{\varvec{b}}}$ by the short-term and fully dynamic scanning protocols. Unfortunately, *k*_4_ was weakly correlated, which may be related to its value being too small and having a high bias to be reliably estimated [33]. In total, short-term PET is closely equivalent to fully dynamic PET for liver kinetic estimation.

Different models have their own advantages and disadvantages. The compartmental model simulates the cavity and constructs a differential model of blood flow conversion and metabolism for parameter fitting. Compartmental models can generally obtain more parameters, reflecting the direction and rate of tracer conversion, and the metabolic process is considered complete. Accurate kinetic modeling of dynamic liver PET data requires consideration of the effect of dual blood supplies in the liver [18]. Moreover, when the fitting calculation introduces some errors, it may affect the fitting of other parameters [34].

The assumption of the maximum-slope method model is based on the blood flow changes before and after the blood flow reaches the peak. The model is simple and convenient for direct calculation with small errors but has fewer parameters [35]. The maximum slope method assumes that there is no tracer outflow in the vein, which is affected by the tracer injection rate [36]. CT perfusion requires a very high injection rate to ensure the accuracy of calculation, but it is not clinically operable [37]. ^18^F-FDG volume is small and the injection is done with a short time, followed by saline for washout, which can enter the cardiopulmonary circulation in a short time, reducing the error generated by calculations. In addition, the compartment model is less affected by the rate of injection [37, 38].

By comparing different three-compartment models, including a single-input model with an input function from the hepatic artery (Model A), a dual-input model with two input functions from the hepatic artery and portal vein (Model B), a single-input model with an input function from the portal vein (Model C), and a dual-input three-compartment model proposed by Wang et al. [18] (Model Wang), Geist et al. [8] suggested that Model Wang has the preferred fitting performance and can be used as a representative of the highest number of fit parameters in the fitted model. Thus, Model Wang was used in this study.

The present study showed that it is feasible to use short-term PET data (5-min dynamic data complemented by 1-min static data at 60 min postinjection) in the liver, analyzed by using a dual-input three-compartment model, for the evaluation of liver kinetic parameters. Three parameters (*k*_2_, *k*_3_ and HPI) were significantly different between HCCs and the background liver tissues (both *P* < 0.05). It is worth noting that an important parameter used to distinguish HCCs from background liver tissues, *K*_1_, was not significantly different (*p* = 0.927). This paper proposes a combined model in which five parameters (*K*_1_, *k*_2_, *k*_3_, HPI, and ${V}_{\mathrm{b}}$) were significantly different between HCCs and background liver tissues (all *p* < 0.05). However, *k*_4_ did not show a significant difference (*p* = 0.896), possibly because the fitted model is still a mathematical calculation. Least squares fitting is used to seek the parameter value when the error of the whole model is minimized, but it is difficult to fit every parameter to the best result because the deviations of some parameters could cause deviations in other parameters.

The hepatic perfusion index (HPI) is an important parameter in assessing liver kinetics. Clinical studies have shown that background liver tissue is supplied with blood primarily from the portal vein, and HCC is mainly supplied by the hepatic artery in vascular proliferation tumors. The HPI of HCC is higher than that of background liver tissue, and the results of both the three-compartment compartment model and the combined model are consistent with this clinical reality. In HCC, the combined model was closer to 80% of the theoretical value than the three-compartment model (76.43% ± 12.09% vs. 71.14% ± 24.56%). Most patients had cirrhosis, which caused increased HPI in background liver tissue. Chandarana et al. [26] reported that the HPI, estimated with a dual-input two-compartment model, was 52.0% ± 23.4% and 12.4% ± 7.1% in cirrhotic and healthy livers, respectively. This paper shows that the HPI of background liver tissue in the combined model is more realistic. (42.76% ± 17.08% vs. 16.78% ± 20.29%).

It is worth noting that most of the kinetic parameters obtained in this study using a short-term protocol with a combined model were consistent with the results estimated with 60-min dynamic PET and different compartment models. According to the study of Geist et al. [8], HCCs had a higher *K*_1_ value in Model Wang (1.95 ± 1.86 vs. 1.90 ± 1.87), a higher *k*_3_ value in Models A–C (0.03 ± 0.02 vs. 0.002 ± 0.006, 0.04 ± 0.03 vs. 0.001 ± 0.001, and 0.03 ± 0.03 vs. 0.001 ± 0.001, respectively) and a lower *k*_*2*_ value in Models A–C and Wang (0.43 ± 0.16 vs. 0.47 ± 0.13, 1.04 ± 0.41 vs. 1.20 ± 1.06, 1.35 ± 0.76 vs. 1.75 ± 1.33, and 0.82 ± 0.73 vs. 1.62 ± 0.90, respectively) than those of background liver tissues. In particular, the *k*_*4*_ value is small and too low to be compared (equal average and lower standard deviation in Model A, lower value in Model Wang).

^18^F-FDG enters tissue cells through glucose transporter proteins on the cell membrane, and it is then phosphorylated by hexokinase in the cell, converted to ^18^F-FDG-6-p, and ultimately retained by the cell; at the same time, these processes are reversible [39]. The expression of glucose transporter proteins is significantly higher in cancer cells than in normal cells. As an important parameter of the compartment model, *K*_1_ reflects the transport rate of blood to tissues and has attracted increased attention from researchers [40]. Wang et al. [18] demonstrated that there is a significant correlation between *K*_1_ and liver inflammation in a study on nonalcoholic fatty liver. They also used *K*_1_ as the main distinguishing criterion in subsequent studies to compare the changes in the three models. Sarkar et al. [41] also concluded that quantitative *K*_1_ is expected to contribute to the noninvasive evaluation of liver inflammation. In the combined model proposed in this study, the *K*_1_ (1.52 ± 0.61 vs. 1.00 ± 0.63) values of HCCs were higher than those of the background liver tissues, which is consistent with previous studies, where glucose transporter protein was higher in HCC than in background liver tissue.

Furthermore, this study showed that *K*_1_ showed a large difference among different patients when using the three-compartment model, while it was relatively consistent when using the combined model (Fig. 5a). One possible reason might be that the dual blood supply to the liver leads to the need for a more accurate dual-input function for the estimation of *K*_1_ [13, 42]. The calculation of HPI in the three-compartment model requires more iterations, which increases the complexity of the model. However, with the combined model, the HPI can be obtained by the maximum slope method and is not involved in the fitting process and considerably improves the appreciation of the other kinetic parameters.

Meanwhile, ^18^F-FDG in the tissues can be cleared into the blood, with *k*_2_ as the clearance rate. Our results show that the *k*_2_ of HCC was higher than that of the background liver tissue in both the dual-input three-compartment model (2.01 ± 0.43 vs. 1.66 ± 0.73) and the combined model (1.84 ± 0.56 vs. 1.01 ± 0.59).*k*_3_ is the rate of phosphorylation, and the expression of hexokinase and its affinity or functional activity for glucose phosphorylation was higher in HCC than in the background liver tissue. Geist et al. [8] found that *k*_3_ was higher in HCC than in background liver tissue in all four different liver kinetic models. Our results are consistent with those of previous studies, and the diagnostic efficacy of *k*_3_ in the combined model (0.046 ± 0.047 vs. 0.005 ± 0.007) was better than that in the dual-input three-compartment model (0.051 ± 0.050 vs. 0.013 ± 0.021).

Hepatocytes contain glucose-6-phosphatase, which is capable of dephosphorylating ^18^F-FDG-6-p to ^18^F-FDG. The results of the experiments in this study showed that *k*_4_, as the rate of dephosphorylation, was higher in background liver tissue than in HCC, indicating that glucose-6-phosphatase activity was higher in background liver tissue than in HCC. However, it was not significantly different in both the dual-input three-compartment model and the combined model, which may be related to the possibility that *k*_4_ values are too small to be accurately estimated.

Compared to the three-compartment model, the combined model allows for many valuable parameters. Our results show that the HAP, TLP and TTP of the combined model could well distinguish between HCCs and background liver tissues, and HAP (91.29 ± 30.70 vs. 32.28 ± 16.71) and TLP (132.46 ± 53.14 vs. 71.18 ± 42.15) values were higher for HCCs than for background liver tissues. Bernstein et al. [15] showed that the TTP of HCCs was lower than that of background liver tissue (17.00 ± 11.60 vs. 47.30 ± 12.80). Hepatocellular carcinoma is mainly nourished by arterial blood flow, and arterial blood flow peaks before portal venous blood flow. This is consistent with our study, where TTP was lower in HCC than in background liver tissue (19.85 ± 12.39 vs. 60.41 ± 59.95).

This study shows that some parameters derived by dynamic PET/CT with pharmacokinetics are better than SUVmax with conventional ^18^F-FDG PET/CT in distinguishing HCC from background liver tissue. Compared to SUVmax, the combined model proposed in this paper provides more perfusion and metabolic information, and it obtains better diagnostic performance for *k*_*3*_ and HPI than SUVmax and is second only to SUVmax for *K*_1_, *k*_2_, and *V*_B_.

In addition, this study assessed the kinetic parameters of liver tissues in HCC patients versus healthy volunteers. The HPI of liver tissues is higher in HCC than in healthy volunteers, which is a result of cirrhosis in HCC patients. Research shows that cirrhosis leads to a decrease in the activity of enzymes in the cells [43, 44], and our results found that *K*_1_, *k*_2_, *k*_3_ and ${V}_{\mathrm{b}}$ were significantly higher in healthy volunteers than in HCC patients.

The goodness of fit (*R*) for nonlinear regression was used to evaluate the TAC fit quality of the different models, with large values indicating a better model [45]. Our results showed that both the three-compartment model and the combined model were applicable for both HCCs and background liver tissues, and the combined model showed a better fit than the three-compartment model, which guaranteed the reliability of the fitting calculation in this study.

This study has some limitations. First, the sample size of the dataset was small. Second, an analysis of the relationship between kinetic parameters and tumor biological characteristics was lacking. Vascular density, hexokinase, glucose transporter and other immunohistochemistry markers were not available in these recruited patients, and the correlation between these immunohistochemistry markers and kinetic parameters should be investigated in future studies. Third, tumor heterogeneity may affect the results, with larger tumor lesions that tend to cause necrosis and hemorrhage in the background tissue, and functional assessment derived from a single region may not reflect perfusion in the liver as a whole. Future studies will further evaluate the whole liver using a pixel-by-pixel method to avoid the impact from ROI delineation. In addition, the reconstruction algorithm may affect the SUV values, but this requires further research into the reconstruction algorithm to improve the image quality of dynamic PET. Finally, further studies are needed to improve the fitting algorithm to satisfy physiological significance and reduce calculation errors.

## Conclusions

This study demonstrated that a short-term protocol (5-min dynamic data complemented by 1-min static data at 60 min postinjection) with a dual-input three-compartment model can be used for estimating liver kinetics and distinguishing HCC from background liver tissue. Compared with conventional ^18^F-FDG PET-derived SUVmax, the kinetic parameters can better distinguish HCC from background liver tissue. Furthermore, this study proposed a combination of the maximum-slope method, and the three-compartment model was preliminarily introduced to enhance the fitting calculation.

## Supplementary Information

**Additional file 1.** **Supplementary Table S1:** ^18^F-FDG PET-derived kinetic parameters in HCCs and background liver tissues by using the maximum-slope model.

## Data Availability

The datasets generated and/or analyzed during the current study are available from the corresponding author on reasonable request.

## Abbreviations

^18^F-FDG: Fluorine-18-fluorodeoxyglucose
CT: Computed tomography
HAP: Hepatic artery perfusion
HCC: Hepatocellular carcinoma
HPI: Hepatic arterial perfusion index
HVP: Hepatic vein perfusion
MR: Magnetic resonance
PET: Positron emission tomography
ROIs: Regions of interest
SUV: Standardized uptake value
SUVmax: Maximum standardized uptake value
TAC: Time-activity curve
TLP: Total liver perfusion
TTP: Time to peak

## References

[1] Yang JD, Hainaut P, Gores GJ, Amadou A, Plymoth A, Roberts LR (2019). A global view of hepatocellular carcinoma: trends, risk, prevention and management. Nat Rev Gastroenterol Hepatol.

[2] Korean Liver Cancer Association (2022) 2022 KLCA-NCC Korea practice guidelines for the management of hepatocellular carcinoma. Korean J Radiol 23(12): 1126–1240. 10.3348/kjr.2022.0822 10.3348/kjr.2022.0822 PMC9747269 36447411

[3] Choi BI, Lee JM (2010). Advancement in HCC imaging: diagnosis, staging and treatment efficacy assessments: imaging diagnosis and staging of hepatocellular carcinoma. J Hepatobiliary Pancreat Sci.

[4] Hennedige T, Venkatesh SK (2016). Advances in computed tomography and magnetic resonance imaging of hepatocellular carcinoma. World J Gastroenterol.

[5] Lu RC, She B, Gao WT (2019). Positron-emission tomography for hepatocellular carcinoma: current status and future prospects. World J Gastroenterol.

[6] Wang SB, Wu HB, Wang QS (2015). Combined early dynamic (18)F-FDG PET/CT and conventional whole-body (18)F-FDG PET/CT provide one-stop imaging for detecting hepatocellular carcinoma. Clin Res Hepatol Gastroenterol.

[7] Jiang HY, Chen J, Xia CC, Cao LK, Duan T, Song B (2018). Noninvasive imaging of hepatocellular carcinoma: from diagnosis to prognosis. World J Gastroenterol.

[8] Geist BK, Wang J, Wang X (2020). Comparison of different kinetic models for dynamic (18)F-FDG PET/CT imaging of hepatocellular carcinoma with various, also dual-blood input function. Phys Med Biol.

[9] Dimitrakopoulou-Strauss A, Pan L, Sachpekidis C (2021). Kinetic modeling and parametric imaging with dynamic PET for oncological applications: general considerations, current clinical applications, and future perspectives. Eur J Nucl Med Mol Imaging.

[10] Winterdahl M, Munk OL, Sørensen M, Mortensen FV, Keiding S (2011). Hepatic blood perfusion measured by 3-minute dynamic 18F-FDG PET in pigs. J Nucl Med.

[11] Samimi R, Kamali-Asl A, Geramifar P, van den Hoff J, Rahmim A (2020). Short-duration dynamic FDG PET imaging: optimization and clinical application. Phys Med.

[12] Sah BR, Leissing CA, Delso G (2018). Evaluation of multifunctional imaging parameters in gastro-oesophageal cancer using F-18-FDG-PET/CT with integrated perfusion CT. Q J Nucl Med Mol Imaging.

[13] Brix G, Ziegler SI, Bellemann ME (2001). Quantification of [(18)F]FDG uptake in the normal liver using dynamic PET: impact and modeling of the dual hepatic blood supply. J Nucl Med.

[14] Wang K, Liu G, Tao Q, Zhai M (2020). Efficient parameters estimation method for the separable nonlinear least squares problem. Complexity.

[15] Bernstine H, Braun M, Yefremov N (2011). FDG PET/CT early dynamic blood flow and late standardized uptake value determination in hepatocellular carcinoma. Radiology.

[16] Sprinz C, Zanon M, Altmayer S, Watte G, Irion K, Marchiori E, Hochhegger B (2018). Effects of blood glucose level on 18F fluorodeoxyglucose (18F-FDG) uptake for PET/CT in normal organs: an analysis on 5623 patients. Sci Rep.

[17] Vita T, Murphy DJ, Osborne MT (2019). Association between nonalcoholic fatty liver disease at CT and coronary microvascular dysfunction at myocardial perfusion PET/CT. Radiology.

[18] Wang G, Corwin MT, Olson KA, Badawi RD, Sarkar S (2018). Dynamic PET of human liver inflammation: impact of kinetic modeling with optimization-derived dual-blood input function. Phys Med Biol.

[19] Huang SC, Phelps ME, Hoffman EJ, Sideris K, Selin CJ, Kuhl DE (1980). Noninvasive determination of local cerebral metabolic rate of glucose in man. Am J Physiol.

[20] Ohashi A, Kataoka M, Kanao S (2019). Diagnostic performance of maximum slope: a kinetic parameter obtained from ultrafast dynamic contrast-enhanced magnetic resonance imaging of the breast using k-space weighted image contrast (KWIC). Eur J Radiol.

[21] Mullani NA, Gould KL (1983). First-pass measurements of regional blood flow with external detectors. J Nucl Med.

[22] Ohno Y, Nishio M, Koyama H, Yoshikawa T, Sugimura K (2012) Dynamic first-pass perfusion area-detector CT analyzed by newly developed and previously applied methods vs dynamic first-pass MRI vs FDG-PET/CT: differential capability of malignant SPN from benign SPN. In: Radiological Society of North America Scientific Assembly & Meeting

[23] Bressem KK, Vahldiek JL, Erxleben C (2019). Comparison of different 4D CT-Perfusion algorithms to visualize lesions after microwave ablation in an in vivo porcine model. Int J Hyperth.

[24] Ohno Y, Fujisawa Y, Yui M, Takenaka D, Koyama H, Sugihara N, Yoshikawa T (2019). Solitary pulmonary nodule: comparison of quantitative capability for differentiation and management among dynamic CE-perfusion MRI at 3 T system, dynamic CE-perfusion ADCT and FDG-PET/CT. Eur J Radiol.

[25] Miyazaki M, Tsushima Y, Miyazaki A, Paudyal B, Amanuma M, Endo K (2009). Quantification of hepatic arterial and portal perfusion with dynamic computed tomography: comparison of maximum-slope and dual-input one-compartment model methods. Jpn J Radiol.

[26] Chandarana H, Block TK, Ream J, Mikheev A, Sigal SH, Otazo R, Rusinek H (2015). Estimating liver perfusion from free-breathing continuously acquired dynamic gadolinium-ethoxybenzyl-diethylenetriamine pentaacetic acid-enhanced acquisition with compressed sensing reconstruction. Invest Radiol.

[27] Passing H, Bablok A (1983). new biometrical procedure for testing the equality of measurements from two different analytical methods. Application of linear regression procedures for method comparison studies in clinical chemistry, Part I. J Clin Chem Clin Biochem.

[28] Liu D, Zhu X, Greenwell B, Lin Z (2023). A new goodness-of-fit measure for probit models: surrogate R(2). Br J Math Stat Psychol.

[29] Maipas S, Nonni A, Politi E, Sarlanis H, Kavantzas NG (2018). The Goodness-of-fit of the fractal dimension as a diagnostic factor in breast cancer. Cureus.

[30] Wang S, Li B, Li P, Xie R, Wang Q, Shi H, He J (2021). Feasibility of perfusion and early-uptake (18)F-FDG PET/CT in primary hepatocellular carcinoma: a dual-input dual-compartment uptake model. Jpn J Radiol.

[31] Kinahan PE, Fletcher JW (2010). Positron emission tomography-computed tomography standardized uptake values in clinical practice and assessing response to therapy. Semin Ultrasound CT MR.

[32] Schierz JH, Opfermann T, Steenbeck J (2013). Early dynamic 18F-FDG PET to detect hyperperfusion in hepatocellular carcinoma liver lesions. J Nucl Med.

[33] Zuo Y, Sarkar S, Corwin MT, Olson K, Badawi RD, Wang G (2019). Structural and practical identifiability of dual-input kinetic modeling in dynamic PET of liver inflammation. Phys Med Biol.

[34] van Herk AM (1995). Least-squares fitting by visualization of the sum of squares space. J Chem Educ.

[35] Lee DH, Lee JM, Klotz E, Han JK (2016). Multiphasic dynamic computed tomography evaluation of liver tissue perfusion characteristics using the dual maximum slope model in patients with cirrhosis and hepatocellular carcinoma: a feasibility study. Invest Radiol.

[36] Mullani NA, Herbst RS, O'Neil RG, Gould KL, Barron BJ, Abbruzzese JL (2008). Tumor blood flow measured by PET dynamic imaging of first-pass 18F-FDG uptake: a comparison with 15O-labeled water-measured blood flow. J Nucl Med.

[37] Kim SH, Kamaya A, Willmann JK (2014). CT perfusion of the liver: principles and applications in oncology. Radiology.

[38] Yang JF, Zhao ZH, Zhang Y (2016). Dual-input two-compartment pharmacokinetic model of dynamic contrast-enhanced magnetic resonance imaging in hepatocellular carcinoma. World J Gastroenterol.

[39] de Prost N, Tucci MR, Melo MF (2010). Assessment of lung inflammation with 18F-FDG PET during acute lung injury. AJR Am J Roentgenol.

[40] Zuo Y, Badawi RD, Foster CC, Smith T, López JE, Wang G (2020). Multiparametric cardiac (18)F-FDG PET in Humans: Kinetic Model Selection and Identifiability Analysis. IEEE Trans Radiat Plasma Med Sci.

[41] Sarkar S, Corwin MT, Olson KA, Stewart SL, Liu CH, Badawi RD, Wang G (2019). Pilot study to diagnose nonalcoholic steatohepatitis with dynamic (18)F-FDG PET. AJR Am J Roentgenol.

[42] Munk OL, Bass L, Roelsgaard K, Bender D, Hansen SB, Keiding S (2001). Liver kinetics of glucose analogs measured in pigs by PET: importance of dual-input blood sampling. J Nucl Med.

[43] Krähenbühl L, Lang C, Lüdes S, Seiler C, Schäfer M, Zimmermann A, Krähenbühl S (2003). Reduced hepatic glycogen stores in patients with liver cirrhosis. Liver Int.

[44] Owen OE, Reichle FA, Mozzoli MA (1981). Hepatic, gut, and renal substrate flux rates in patients with hepatic cirrhosis. J Clin Invest.

[45] Wu J, Zhao C (2019) Cooperation on the Monte Carlo Rule: prisoner’s dilemma game on the grid. In: Theoretical computer science. Springer, Singapore

