# Supplementary material for: Short-term PET-derived kinetic estimation for the diagnosis of hepatocellular carcinoma: a combination of the maximum-slope method and dual-input three-compartment model
Source: Insights Imaging. 2023 May 24;14:98. doi: 10.1186/s13244-023-01442-5 (PMC10209370; doi:10.1186/s13244-023-01442-5)
Supplement: Supplementary file 1 — Additional file 1. Supplementary Table S1: 18F-FDG PET-derived kinetic parameters in HCCs and background liver tissues by using the maximum-slope model. [file 13244_2023_1442_MOESM1_ESM.pdf]

Short-term PET-derived kinetic estimation for the diagnosis of hepatocellular carcinoma: A combination of the maximum-slope method and dual-input three-compartment model

Supplementary Table S1.

^{18}F-FDG PET-derived kinetic parameters in HCCs and background liver tissues by using the maximum-slope model.

**Parameters**	**HCCs** n=24	**Liver tissues** n=21	**AUC**	***P***
**HAP** (ml/100 ml/min)	91.29±30.70	32.38±16.71	0.987	<0.001
**HVP** (ml/100 ml/min)	37.55±24.10	44.75±38.74	0.500	0.462
**TLP** (ml/100 ml/min)	132.46±53.14	71.18±42.15	0.861	<0.001
**TTP** (s)	19.85±12.39	60.41±59.95	0.827	0.002
